# Clinical Report from Medical Wards of Mercy Hospital

**Published:** 1869-02

**Authors:** N. S. Davis

**Affiliations:** Professor of Practical and Clinical Medicine, in Chicago Medical College


### The Clinique.

### CLINICAL REPORT FROM MEDICAL WARDS OF MERCY HOSPITAL.

By N. S. DAVIS, M.D., Professor of Practical and Clinical Medicine, in Chicago Medical College.

Reported by W. A. Barstow.

Gentlemen:—I shall occupy your attention during the clinic hour with a case of cardiac disease. Upon auscultation, we have no difficulty in locating the disease in the heart; but when we go back of that, and try to ascertain what particular valves are involved, it is not so easy.

You will observe the patient is in a sitting posture, which seems to be his most comfortable position.

The flush on the face and hands is not due to fever, as there is no perceptible increase of heat of the surface; but it is due to capillary congestion, which is sometimes of a purplish hue. If the patient were to go down-stairs and return, or take any active exercise, the flush would become decidedly more venous, as well as the respiration more labored and oppressed. There will be noticed above the clavicles slight pulsation in the jugulars, which is also much increased on exercise.

You could not, under any circumstances, have a patient in a more favorable condition to distinguish the exact condition of the heart's action, as he has been taking arterial sedatives for several days, and is now sufficiently under the influence of the medicine to render the sounds very distinct. A little below and to the left of the nipple, upon the application of the stethoscope, you will get a double murmur: one harsh, rasping, and loud, the other softer and shorter. The first is synchronous with the systole, and the second with the diastole. The first is heard over the whole cardiac space, the second only over the left side. This shows that the principal difficulty is in the auriculo-ventricular opening of the left side—the location of the mitral valves.

You hear the rasping sound all about the region of the heart; but move which way you will, when you return a little to the left and below the nipple, you hear it the loudest. The second sound you hear is due to slight regurgitation. Over the rest of the heart you get only one sound, occupying the entire time of both sounds of the heart. The hypertrophy in this case is well marked. When the patient was not under the effects of medicine, there was regurgitation on the right side, giving a strong pulsation in the epigastric region through the ascending vena cava, and also the descending vena cava to the jugulars.

When I first examined the patient, there seemed to be a well-marked aneurismal tumor in the epigastric region, which was quite tender to the touch. This tumor was present two days ago, when I called the attention of the other division of the class to it, but I see it is absent to-day.

This man's heart is probably in the same condition as that of the man who died several weeks since, and whose heart I exhibited to you—*viz.:* a dilatation of the right ventricle; contraction of the mitral opening, with thick, roughened edges, and preventing a complete closure during the systole of the ventricles; and general hypertrophy of the muscular structure.

When the patient is disturbed, the tricuspid valve is insufficient to close the auriculo-ventricular opening of the right side, because it is enlarged by the dilatation of the right ventricle. The apparent pulsating tumor in the right side of the epigastrium was probably caused by overfulness of the vena portæ, from regurgitation through the auriculo-ventricular opening of the right side into the ascending vena cava.

If you notice the feet and ankles, they will be found quite œdematous; a condition which is present in most cases of cardiac disease in the advanced stages. If the pathological conditions of the patient have been described correctly, the prognosis is unfavorable; and to render the action of the heart slower and more uniform, constitute the principal objects of treatment. Just in proportion as they can be accomplished, will the patient be rendered more comfortable, and his life be prolonged. By rendering the action of the heart slower and more uniform, more time will be allowed after each systole for the blood to pass through the narrowed mitral opening from the left auricle; and hence the pulmonary circulation will be less obstructed, and the equilibrium between the fulness of the right and left cavities of the heart be better maintained.

To fulfil the indications just stated, the patient is taking one fluid drachm of the following prescription before each meal and at bedtime:—

℞. Fluid Ext. Scutellaria,---------------- ʒiij.

" " Digitalis,------------------ ʒj.

Mix.

Also, five grains of Sub. Nit. Bismuth, half an hour after each meal.

When the patient commenced the use of the scutellaria and digitalis, four days since, his pulse was 110 per minute, and irregular, with dyspnœa, epigastric distress, cool and purplish-colored extremities, and strong pulsation in the jugular and subclavian veins.

Now his pulse is only 60 per minute, and regular, with a decided improvement in all his symptoms. His diet has been plain and nutritious. The same treatment will be continued, with careful attention, to prevent any excess in the action of the digitalis.

